# Cloning, overexpression, purification, and characterization of a polyextremophilic β-galactosidase from the Antarctic haloarchaeon *Halorubrum lacusprofundi*

**DOI:** 10.1186/1472-6750-13-3

**Published:** 2013-01-16

**Authors:** Ram Karan, Melinda D Capes, Priya DasSarma, Shiladitya DasSarma

**Affiliations:** 1 Department of Microbiology and Immunology, University of Maryland School of Medicine, and Institute of Marine and Environmental Technology, University System of Maryland, 701 E Pratt Street, Baltimore, MD, 21202, USA

**Keywords:** Polyextremophiles, Extremozymes, Protein stability, Halophiles, Psychrophiles, Biofuels

## Abstract

**Background:**

*Halorubrum lacusprofundi* is a cold-adapted halophilic archaeon isolated from Deep Lake, a perennially cold and hypersaline lake in Antarctica. Its genome sequencing project was recently completed, providing access to many genes predicted to encode polyextremophilic enzymes active in both extremely high salinity and cold temperatures.

**Results:**

Analysis of the genome sequence of *H. lacusprofundi* showed a gene cluster for carbohydrate utilization containing a glycoside hydrolase family 42 β-galactosidase gene, named *bga*. In order to study the biochemical properties of the β-galactosidase enzyme, the *bga* gene was PCR amplified, cloned, and expressed in the genetically tractable haloarchaeon *Halobacterium* sp. NRC-1 under the control of a cold shock protein (*csp*D2) gene promoter. The recombinant β-galactosidase protein was produced at 20-fold higher levels compared to *H. lacusprofundi*, purified using gel filtration and hydrophobic interaction chromatography, and identified by SDS-PAGE, LC-MS/MS, and ONPG hydrolysis activity. The purified enzyme was found to be active over a wide temperature range (−5 to 60°C) with an optimum of 50°C, and 10% of its maximum activity at 4°C. The enzyme also exhibited extremely halophilic character, with maximal activity in either 4 M NaCl or KCl. The polyextremophilic β-galactosidase was also stable and active in 10–20% alcohol-aqueous solutions, containing methanol, ethanol, *n*-butanol, or isoamyl alcohol.

**Conclusion:**

The *H. lacusprofundi* β-galactosidase is a polyextremophilic enzyme active in high salt concentrations and low and high temperature. The enzyme is also active in aqueous-organic mixed solvents, with potential applications in synthetic chemistry. *H. lacuprofundi* proteins represent a significant biotechnology resource and for developing insights into enzyme catalysis under water limiting conditions. This study provides a system for better understanding how *H. lacusprofundi* is successful in a perennially cold, hypersaline environment, with relevance to astrobiology.

## Background

The genome sequence of a cold-adapted extremely halophilic archaeon, *Halorubrum lacusprofundi*, was recently completed and analyzed by comparative genomics [1-3]. Like other halophilic Archaea, *H. lacusprofundi* has the ability to grow and thrive in salt-rich environments and its metabolic processes and physiological functions prevail under nearly saturating salt conditions similar to those found in their natural hypersaline environment (21–28%, w/v salt content) [4-6]. Among halophiles, *H. lacusprofundi* is distinguished by survival in a perennially cold habitat, Deep Lake, Antarctica, where the normal temperature is in the range from +11.5°C to −18°C. The lake brine remains liquid throughout the year as a result of freezing point depression from the extremely high salinity. Due to its success in such an unusually harsh environment, *H. lacusprofundi* has been of significant microbiological, biotechnological, and astrobiological interest [5,7-9].

With the release of the *H. lacusprofundi* genome sequence, we identified the *bga* gene coding a glycoside hydrolase. The *bga* gene product is a putative β-galactosidase (E.C. 3.2.1.23), well-known to hydrolyze lactose into glucose and galactose. Broadly, β-galactosidases are classified into seven glycoside hydrolase (GH) families based on functional similarities [10], with the extensively studied *E. coli* enzyme belonging to the GH-2 family [11]. Extremophilic β-galactosidases from cold-adapted, halophilic, and thermophilic species are members primarily of two families, either the GH-2 or GH-42 family [12-17]. The *H. lacusprofundi* β-galactosidase has been classified as a member of the GH-42 family [10].

**Figure 1:**
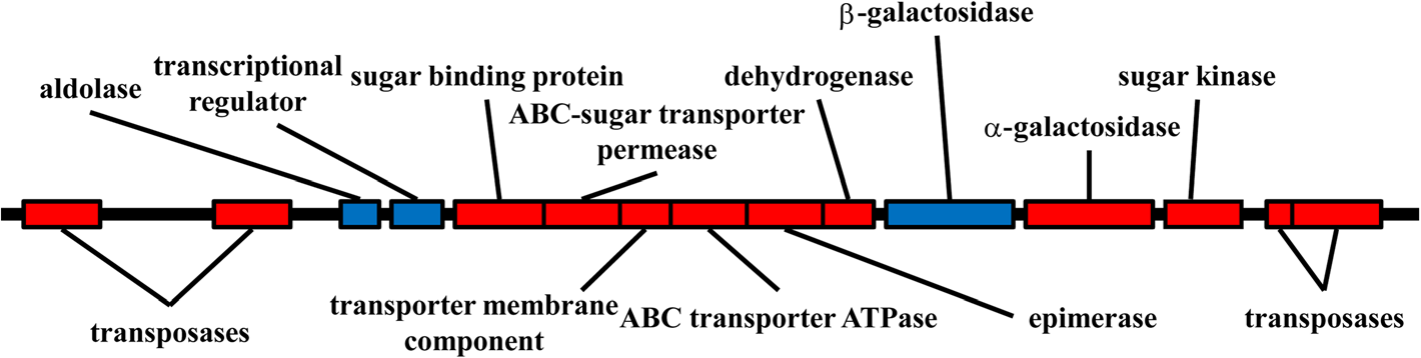
***H. lacusprofundi *carbohydrate utilization gene cluster in chromosome II.** The predicted genes, shown to scale, with those in rightward transcriptional orientation in red and leftward orientation in blue. Predicted gene functions are (from left to right) Hlac_2855 and 2857, ISH3 and IS4 family transposases; Hlac_2860, 2-keto-3-deoxy-6-phosphogluconate aldolase/4-hydroxy-2-oxoglutarate aldolase; Hlac_2861, IclR family transcriptional regulator; Hlac_2862, sugar-binding periplasmid protein; Hlac_2863, ABC-type sugar transport system permease component; Hlac_2864, sugar permease; Hlac_2865, ABC-type sugar transport system; ATPase component; Hlac_2866, L-alanine-DL-glutamate epimerase and related enzymes of enolase; Hlac_2867, short-chain dehydrogenase/reductase SDR; Hlac_2868, β-galactosidase; Hlac_2869, α-galactosidase; Hlac_2870, sugar kinase, and Hlac_2871 and 2872, IS200 family and IS605 OrB family transposases.

The biological function of β-galactosidases from environmental and extremophilic microorganisms has been the subject of several recent investigations [18-21]. In environments where lactose is not normally available, β-galactosidase enzymes may act on short chain oligosaccharides released from pectin galactans. Enzymes capable of degrading the larger polymers are encoded nearby in the genomes of several microorganisms. In the *H. lacusprofundi* genome, a gene cluster on chromosome II is present, containing genes for sugar-binding periplasmic proteins, ABC sugar transporter system, α and β-galactosidases, and a kinase (Figure 1). The *H. lacusprofundi* β-galactosidase, together with the products of nearby genes, likely functions in the breakdown of plant polymers and utilization of galactose via the De Ley-Doudoroff pathway [3,21]. Interestingly, the gene cluster is flanked by predicted transposases, suggestive of a mobile genetic element.

The *H. lacusprofundi* β-galactosidase enzyme was of interest as a result of its expected “polyextremophilic” character with predicted activity at both high salt concentrations and extreme temperatures. Such polyextremophilic enzymes may have novel biotechnological applications, for example in synthetic chemistry, where they can be active and stable in the presence of organic solvents due to tight binding of water [22]. Organic solvents in reaction mixtures increase the solubility of hydrophobic substrates, and have the potential to improve the kinetic equilibrium and increase the yield and specificity of the product [23]. However, organic solvents generally disrupt hydrophobic interactions within enzymes, causing them to lose their catalytic activity. High salt solutions and low temperatures mimic non-aqueous environments, since water activity is reduced and enzymes must successfully compete for available water for function [8].

In the present study, we have purified and characterized the GH-42 family β-galactosidase from the cold-adapted haloarchaeon, *H. lacusprofundi*, after overexpression of the gene in the model haloarchaeon, *Halobacterium* sp. NRC-1. Our results show that the enzyme is active at high salinity and wide temperature range, and functions in the presence of a number of organic solvents.

## Methods

### Materials

Restriction enzymes, T4 DNA ligase, and Taq DNA polymerase were purchased from New England Biolabs (Beverly, MA, USA). X-Gal (5-bromo-4-chloro-3-indolyl-β-D-galacto-pyranoside) and ONPG (*o*-nitrophenyl-β-D-galactopyranoside) were purchased from IBI Scientific (Peosta, Iowa, USA) and USB Corporation (Cleveland, OH, USA). All other chemicals were purchased from Sigma (St. Louis, MO, USA).

### Strains, media, and culture conditions

*Halorubrum lacusprofundi* isolated from Deep Lake, Antarctica [4] was obtained from the American Type Culture Collection (ATCC) (strain ATCC 49239). It was grown in ATCC medium 1682, artificial Deep Lake medium, prepared according to the directions from ATCC at 30°C with shaking. *Escherichia coli* DH5α was grown at 37°C in Luria-Bertani (LB) medium supplemented with 100 μg/ml ampicillin. *Halobacterium* sp. strain NRC-1 and derivatives were cultured in CM^+^ medium containing 4.3 M NaCl and trace metals at 42°C with shaking as previously described [24,25]. For solid media, 2% (w/v) agar was added, and when required, 5-bromo-4-chloro-indolyl-β-D-galactopyranoside (X-Gal) was added to 40 μg/ml. Stock cultures were maintained in glycerol at −80°C. For short-term use, purified cultures were maintained on stock plates at 4°C.

### Measurement of β-galactosidase activity

Cells were harvested by centrifugation (6,000 × g, 4°C, 10 min) in a Sorvall RC-5B centrifuge and disrupted in 50 mM Tris-buffer, pH 8.0 (containing 2 M KCl, 10% v/v glycerol, 0.1 mM ZnCl_2_, 50 mM MgCl_2_, 1 mM EDTA, and 100 μg PMSF/ml) using a sonicator (Model CL-18, Fisher Scientific, USA). Cell debris was removed by centrifugation (25,000 × g, 4°C, 10 min) in an Eppendorf 5417C centrifuge to obtain the crude extract and analyzed for β-galactosidase activity. Enzymatic activity was carried out for 10 minutes at 25°C and pH 6.5 using 2.2 mM of the synthetic chromogenic substrate *o*-nitrophenyl β-D galactopyranoside (ONGP) as a substrate and stopped by the addition of Na_2_CO_3_ to 1.0 M concentration [18,26]. The *o*-nitrophenol released from ONPG by β-galactosidase was measured at 420 nm using a UV-1601 spectrophotometer (Shimadzu, Kyoto, Japan). One international unit (IU) of β-galactosidase activity is defined as the amount of enzyme liberating 1 μmol of *o*-nitrophenol per minute. Miller units (MU) are calculated as 1,000 × OD_420_ / (*t* × V × OD_600_), where *t* is the reaction time in minutes, V is the volume of cells used in milliliters and cell density is measured at OD_600_[27].

### Bioinformatic analysis

Bioinformatic analysis of the *H. lacusprofundi* genome was conducted using tools on the Carbohydrate Active Enzymes (CAZY) database (http://www.cazy.org) and the HaloWeb site (http://halo4.umbi.umd.edu) [10,28]. The HaloWeb site provided access to the *H. lacusprofundi* genome information, including genetic maps, gene functions, DNA and protein sequences, and haloarchaeal orthologous groups (HOGs) [2,10,28]. The β-galactosidase gene (Hlac_2868) and surrounding genes were further analyzed using NCBI clusters of orthologous groups (COGs) [29]. Similar proteins were also identified by BlastP analysis using the *H. lacusprofundi* predicted protein sequence as query and downloaded for local analysis from NCBI [30,31]. The CAZY database provided the assignment of the *H. lacusprofundi* β-galactosidase protein as a glycosyl hydrolase family 42 member and links to additional information, including homologous enzymes and structures. Additional phylogenetic analysis of β-galactosidase protein sequences was performed using ClustalX [32].

### Construction of the β-galactosidase gene expression plasmid

**Figure 2:**
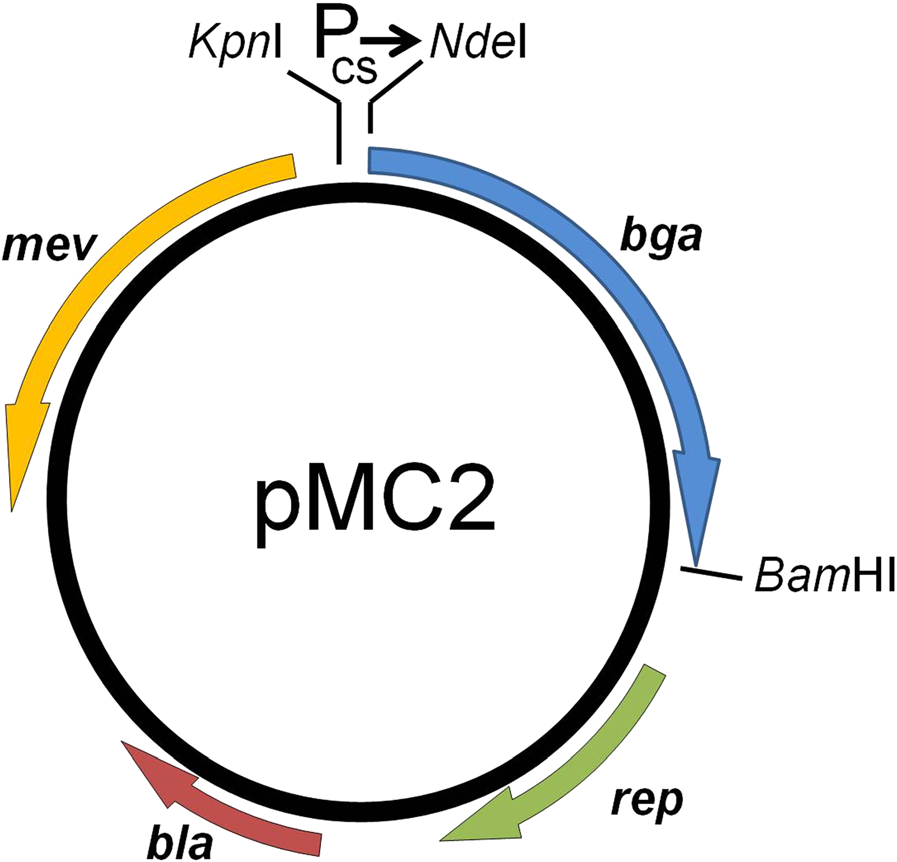
**Shuttle vector pMC2 used for the expression of *H. lacusprofundi bga *in *Halobacterium *sp. NRC-1.** Location and transcriptional orientation of *bla*, β-lactamase for ampicillin resistance; *mev*, HMG-CoA reductase for mevinolin resistance; *bga*, β-galactosidase, and *rep*, the *Halobacterium* pGRB replicase gene are shown. Position of the temperature induced promoter, Pcs→ and *Kpn*I, *Nde*I and *Bam*HI restriction sites are indicated.

To facilitate protein expression in haloarchaea, an overexpression vector was constructed. On the basis of prior heat-shock and cold-adaptation microarray results [33], the *Halobacterium* sp. NRC-1 *csp*D2 promoter was selected for fusion to the *H. lacusprofundi* β-galactosidase gene. First, a 103 bp PCR fragment containing the *csp*D2 promoter was cloned into the *E. coli-Halobacterium* sp. NRC-1 shuttle vector, pKJ408 (gift from Dr. Kevin Jung and John L. Spudich, University of Texas Health Sciences Center, Houston) using *Kpn*I and *Nde*I sites, resulting in an intermediate vector, pMC1. Next, the β-galactosidase (*bga*) gene from *H. lacusprofundi* was PCR amplified from the genome and cloned, via *Nde*I and *Bam*HI sites into pMC1, to generate the pMC2 expression plasmid (Figure 2). The construct was validated by restriction digestion using *Kpn*I, PCR amplification, and DNA sequencing. Primers used for amplification and sequencing are listed in Table 1.

**Table 1:** Oligonucleotides used in this study as primers for PCR

**Primer**	**5**^**′**^**-3**^**′**^**sequence**	**Use**
cspD2F	CGGGGTACCGGCACTGCACAGCCGATGCT	Amplification of *csp*D2 promoter
cspD2R	GGAATTCCATATGCAATCAACCGTTGGGCCG	
HlaR	CGGGATCCCTACGTCTGAGCGATTCT	Amplification of *bga* gene
HlaF	GGGAATTCCATATGCGTCTCGGAGTCTG	
β-gal 05R	TCGAAGGAGCCGTACTGCTG	Sequencing of *bga* gene
β-gal 15 F	CCGTCGATCAGCACGAGTCG	
β-gal 04 F	GATTGTGCCGACGCGTTCCG	
β-gal 16R	TCCAGCCACTCCGCCCAGGT	
β-gal 11R	TCCTCGGACGTGTGTGCGGC	
β-gal l10F	GCACCACGCCGCCGCCCACG	
pKJprmhtrF	TTATTCCGTTTCCGCGGAAA	Sequencing of *csp*D2 promoter in pMC2 plasmid
pKJprmhtrR	GTCGTCCTCGTTGTCCCCGA	

### Expression of the β-galactosidase gene in *Halobacterium* sp. NRC-1

*Halobacterium* sp. NRC-1, which does not possess an endogenous *bga* gene, was transformed with pMC2, using the EDTA-PEG method [24] and transformants were selected on CM^+^ agar plates supplemented with 20 μg/ml mevinolin. Transformants were either grown to late log phase (OD_600_ of 0.9–1.0) at 42°C in CM^+^ medium supplemented with 20 μg/ml mevinolin or streaked on CM^+^ plates containing 40 μg/ml X-Gal and 20 μg/ml mevinolin. To induce β-galactosidase production, the cultures were further incubated at various temperatures for 72 h.

### β-galactosidase purification

Crude cell extract (2 ml), prepared as described above, was subjected to gel filtration chromatography on a Sephadex G-200 (Pharmacia Fine Chemicals, Sweden) column (2.5 cm × 50 cm) equilibrated with 50 mM sodium phosphate buffer, pH 7.0, containing 1.25 M (NH_4_)_2_SO_4_ and 1.25 M KCl. Fractions of 2.0 ml were collected at a flow rate of 0.35 ml/min using a fraction collector (Bio-Rad Laboratories, Hercules, CA) using the same buffer. Protein content (Abs 280 nm) and β-galactosidase activity were determined for collected fractions. The active fractions obtained by gel filtration were combined and further purified by hydrophobic interaction chromatography (HIC) on a Phenyl Sepharose 6 Fast Flow (Sigma, St. Louis, MO, USA) column (1.5 cm × 50 cm), equilibrated with 50 mM sodium phosphate buffer, pH 7.0 containing 1.25 M (NH_4_)_2_SO_4_ and 1.25 M KCl. After loading 25 ml of sample, the column was washed with the same buffer until unbound proteins were removed. The bound proteins were eluted with a decreasing gradient (100–0%) of 50 mM sodium phosphate buffer, pH 7.0, containing 1.25 M (NH_4_)_2_SO_4_ and 1.25 M KCl and increasing gradient (0–100%) of 50 mM sodium phosphate buffer, pH 7.0. Fractions (2.0 ml each) were collected at a flow rate of 0.35 ml/min using a fraction collector and analyzed for protein concentration and β-galactosidase activity. Protein concentrations were determined by the Bradford dye binding method [34] using bovine serum albumin as a standard. During chromatographic purification steps, protein concentration was estimated by recording the absorbance at 280 nm with a BioLogic LP system (Bio-Rad Laboratories, Hercules, CA).

### Polyacrylamide gel electrophoresis

Sodium dodecyl sulphate polyacrylamide gel electrophoresis (SDS-PAGE) was carried out according to the method of Laemmli [35] using 8% (29:1 acrylamide to bis-acrylamide) cross-linked polyacrylamide gels on a vertical gel electrophoresis unit (Bio-Rad Laboratories). The gels were stained with 0.1% Coomassie blue R-250 (in methanol–acetic acid–water, 40:10:50, v/v) followed by destaining with methanol–acetic acid–water (40:10:50, v/v).

### Identification of purified protein by LC-MS/MS analysis

To facilitate identification of purified protein by LC-MS/MS analysis, the Coomassie-stained protein band was excised and disulfide bonds were reduced with tris(hydroxypropyl)phosphine (TCEP). Next, the protein was digested with trypsin and peptides were separated by nanoscale reverse-phase liquid chromatography using an Xtreme Simple nanoLC system (CVC/Micro-Tech, Fontana, CA). A LTQ-Orbitrap mass spectrometer (Thermo Electron, Waltham, MA) equipped with a nanospray ionization source was used for data generation. MS/MS spectra were searched against protein databases using Sorcerer™-SEQUEST® (Sage-N Research, Milpitas, CA). The quality of peptide and protein assignments was assessed using PeptideProphet and ProteinProphet [36].

### Effect of salt, pH, and temperature on β-galactosidase activity

The effect of NaCl/KCl concentration on β-galactosidase activity was evaluated in the presence of 0–4.5 M NaCl/KCl in the enzyme reaction mixture. The effect of pH was evaluated by assaying β-galactosidase activity in 50 mM sodium phosphate (pH 6.0–7.0) or Tris–HCl (pH 7.5–8.0) buffers. A plot of relative activity against pH was created to determine the optimum pH for the reaction. To determine the optimum temperature, the activity of β-galactosidase was measured at various temperatures. The percentage of maximal activity was calculated by considering the maximum activity under the given conditions as 100%.

### Effect of organic solvents on the activity and stability of β-galactosidase

To determine the effect of organic solvents on β-galactosidase activity, enzyme assays were performed in the absence and presence of organic solvents (5 and 10%, v/v methanol, ethanol, *n*-butanol, and isoamyl alcohol). For the stability of the purified β-galactosidase in aqueous-alcohol solutions, enzyme was pre-incubated at 30°C with constant shaking at 200 rpm for 3 h in the absence or presence of organic solvent (10 and 20%, v/v). Samples were taken at different time intervals and the residual enzyme activity was determined as described above.

## Results

### *H. lacusprofundi* β-galactosidase gene, protein, and enzyme activity

**Figure 3:**
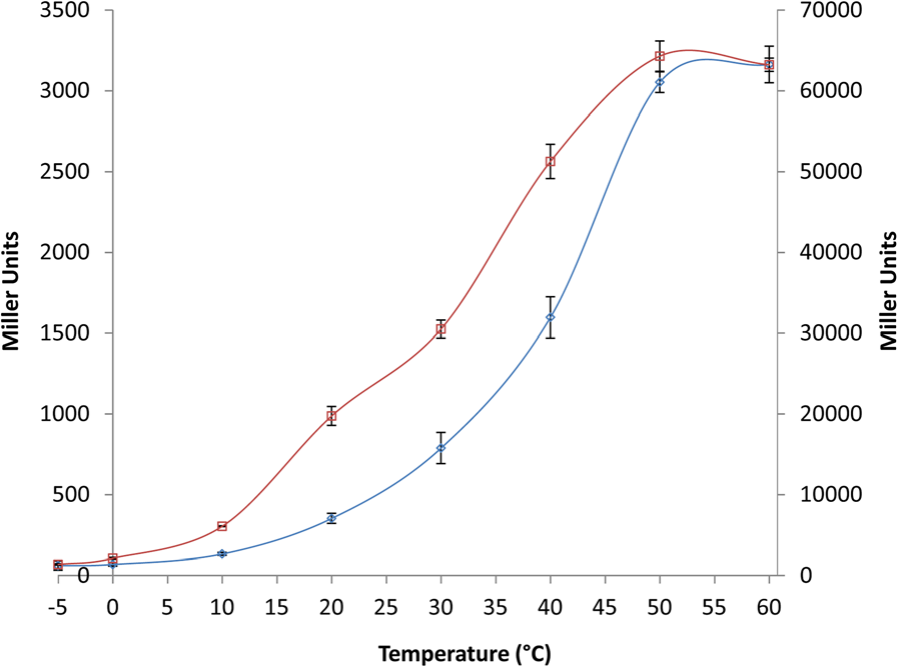
**β-galactosidase activity measured in crude extracts of wild-type *H. lacusprofundi *(blue, ◊) and *Halobacterium *sp. NRC-1 (pMC2) (red, □) at different temperatures.** The enzyme activity in Miller units (MU) for the former haloarchaeon is shown on the left and the latter is shown on the right. Values are the average of triplicate experiments with standard deviation shown with error bars.

The β-galactosidase gene (Hlac_2868, *bga*) was identified during annotation of the genome of *H. lacusprofundi* in a region of chromosome II containing a gene cluster for the binding, uptake, and utilization of mono- and oligosaccharides (Figure 1) [1-3]. The β-galactosidase gene contains an open reading frame of 2,100 bp which encodes a protein of 700 amino acid residues with a predicted molecular mass of 78.06 kDa. Typical of haloarchaeal proteins, the *bga* gene product contains a high percentage (18.6%) of acidic residues and a predicted acidic pI of 4.4 [37]. To determine if this gene was expressed into an active β-galactosidase enzyme, we tested whether *H. lacusprofundi* forms blue colonies when plated on agar plates supplemented with the chromogenic substrate, X-gal. Since blue colonies were indeed observed (data not shown), we proceeded to assay for breakdown of ONPG in crude extracts of *H. lacusprofundi* (Figure 3). β-galactosidase activity was readily observed from −5°C to 60°C. The high salt concentration of the lysates resulted in freezing point depression and allowed for measurement of enzyme activity at subzero temperatures, which showed that the enzyme is able to function at −5°C, albeit with low efficiency (~ 2–5% of optimal).

### Cloning and overexpression of *H. lacusprofundi* β-galactosidase gene in *Halobacterium* sp. NRC-1

In order to study the *H. lacusprofundi* β-galactosidase in more detail, we cloned and overexpressed the *bga* gene in a genetically tractable haloarchaeal host, *Halobacterium* sp. NRC-1, which lacks an endogenous β-galactosidase [38]. The expression plasmid, pMC2, contained the β-lactamase gene (*bla*) for selection of ampicillin resistance in *E. coli*, HMG-CoA reductase gene (*mev*) for selection of mevinolin resistance in *Halobacterium* sp. NRC-1, and origins of replication for both *E. coli* (ColE1 plasmid) and *Halobacterium* sp. NRC-1 (pGRB plasmid) (Figure 2) [39,40]. Plasmid pMC2 was transformed into *Halobacterium* sp. NRC-1, and plated on CM^+^ agar plates containing X-gal and mevinolin. β-galactosidase enzyme activity was determined by the appearance of blue NRC-1 colonies on agar plates (data not shown). The *Halobacterium* sp. NRC-1(pMC2) strain was grown in liquid culture, lysed, and crude lysate assayed for β-galactosidase activity (Figure 3). The results clearly demonstrated that the recombinant *Halobacterium* sp. NRC-1(pMC2) strain produces high levels of β-galactosidase, nearly 20-fold higher than wild-type *H. lacusprofundi* (Figure 3). A nearly identical temperature profile was observed for the enzyme produced in both *H. lacusprofundi* and *Halobacterium* sp. NRC-1(pMC2), with activity from −5°C to 60°C.

**Figure 4:**
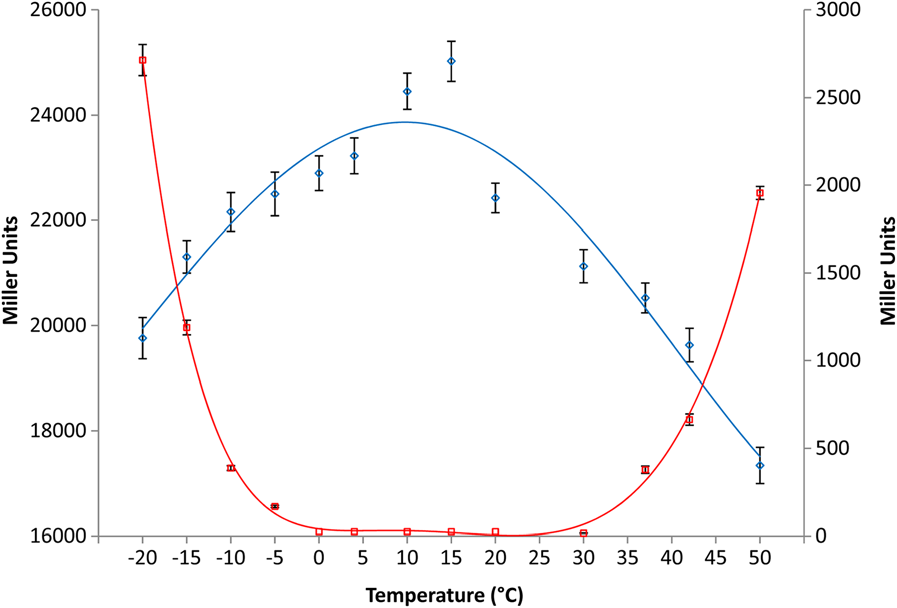
**Induction of *H. lacusprofundi *β-galactosidase in *Halobacterium *sp. NRC-1 (pMC2).** Enzyme assays were done at 25°C after incubating cells for 72 hours at various temperatures for cell lysate (blue, ◊) and supernatant (red, □). Activity levels are shown in Miller units (MU) on the left for cell lysate and the right for supernatant, and are the average of triplicate experiments, with standard deviation shown with error bars.

Next, induction of *H. lacusprofundi* β-galactosidase produced in *Halobacterium* sp. NRC-1(pMC2) at different temperatures was monitored (Figure 4). Cultures were incubated for 72 hours at different temperatures from −20 to 70°C and β-galactosidase activity assayed in cell lysate and supernatant. Maximum enzyme activity was observed with induction at 15°C, consistent with previous transcriptomic data for expression of the *csp*D2 gene (Figure 4). Substantial β-galactosidase activity was observed in the supernatant at both high and low temperature extremes (−10 to −20°C and 40 to 50°C), likely due to cell-lysis at these temperatures.

### Purification and identification of *H. lacusprofundi* β-galactosidase

**Figure 5:**
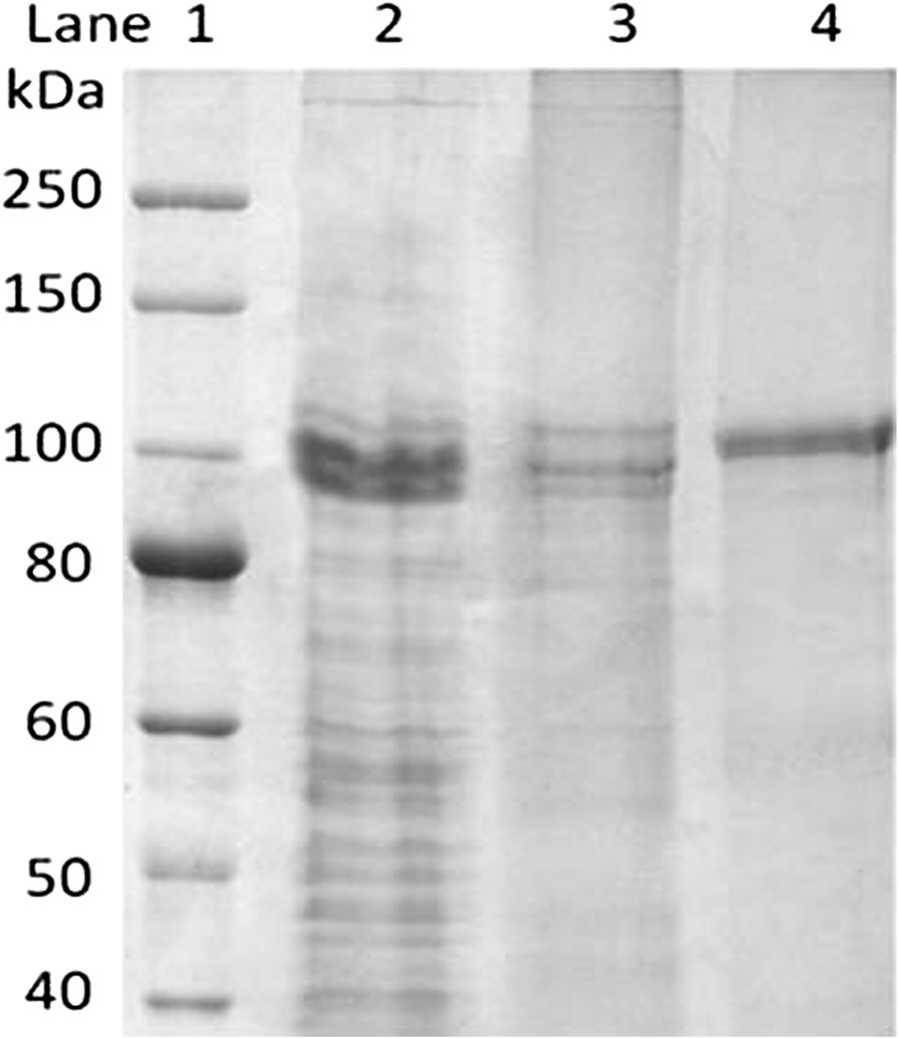
**Purification of *H. lacusprofundi *β-galactosidase overproduced in *Halobacterium *sp. NRC-1.** Proteins are shown electrophoresed on an 8.0% polyacrylamide gel after Coomassie blue staining: Lane 1: Molecular mass markers; Lane 2: Cell lysate; Lane 3: Gel filtration active fractions; Lane 4: HIC active fractions.

The *H. lacusprofundi* β-galactosidase was purified by a combination of gel-filtration and hydrophobic interaction chromatography, in the presence of high concentrations of salt, and identified by LC-MS/MS analysis (Table 2). Gel-filtration chromatography of cell lysate led to 4.9-fold purification with 18 units/mg protein specific activity and 80% yield. HIC further enhanced the specific activity to 111 units/mg protein with 30-fold purification and 18% yield. Subsequent SDS-PAGE analysis of the HIC fractions revealed a highly prominent band corresponding to a peptide with a molecular mass of about 100 kDa (Figure 5). The higher apparent molecular mass was expected based on previous results with haloarchaeal proteins [41]. To validate the identity of the protein, the band was excised from the gel, subjected to trypsin digestion, and analyzed by LC-MS/MS. MS/MS spectra were searched against a protein database using Sorcerer™-SEQUEST®. Thirteen unique peptides corresponding to *H. lacusprofundi* β-galactosidase sequence were observed, confirming the identity of the protein (Table 3).

**Table 2:** **Purification of *H. lacusprofundi *** **β-galactosidase**

**Purification step**	**Total protein (mg)**	**Total activity (IU)**	**Yield (%)**	**Specific activity (units/mg protein)**	**Fold purification**
Cell lysate	7.92	29.84	100.00	3.76	1.00
Gel filtration	1.31	23.99	80.42	18.31	4.87
Hydrophobic interaction chromatography	0.048	5.32	17.82	110.83	29.47

**Table 3:** Unique peptides identified by LC-MS/MS analysis

**Amino acid sequence**	**Number of times peptide identified**	**Expected mass value (Da)**	Query coverage* (%) to *H. lacusprofundi * **β-galactosidase**	**Identity with *H. lacusprofundi *** **β-galactosidase**
DVAAMADAGLEYVR	1	1480.6	3	100
FSSDSVVEYNR	1	1302.3	1	100
HYCFNSDAYR	2	1275.3	1	100
TWVTNFTSDR	1	1226.3	2	100
WLVDERPSIR	1	1270.4	1	100
YDGEASPDQLR	1	1250.2	1	100
LHADLIR	1	836.9	1	100
TGVKDAENK	5	961.0	1	100
AGDPDQVGMDHDIYR	1	1688.7	3	100

* The percent of *H. lacusprofundi* β-galactosidase amino acid sequence that aligns with the identified peptide sequence.

### Characterization of purified *H. lacusprofundi* β-galactosidase

**Figure 6:**
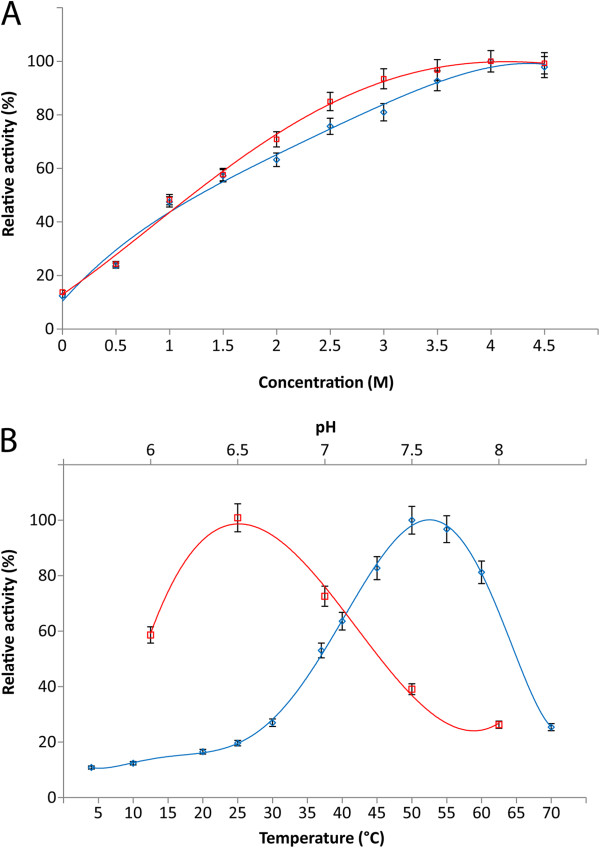
**Properties of purified *H. lacusprofundi *β-galactosidase.** Effect of various parameters on enzyme activity: (A) varying KCl (blue, ◊), NaCl (red, □) concentrations, measured at 37°C and pH 6.5, (B) varying pH (red, □), measured at 37°C and 4.0 KCl, and varying temperature (blue, ◊), measured at pH 6.5 and 4.0 KCl. Enzyme activity (%) was defined as the percentage of activity detected with respect to the maximum observed β-galactosidase activity in each series. Values are the average of triplicate experiments with standard deviation shown with error bars.

The purified β-galactosidase enzyme preparation was assayed for activity over a broad temperature range and KCl and NaCl concentrations. The results were similar with either NaCl or KCl, tested up to 4.5 M, with maximum activity found at 4.0-4.5 M with either salt (Figure 6A). The enzyme is most active at the high salt concentrations similar to that reported intracellularly in haloarchaea. For temperature activity, the enzyme was assayed from 4–70°C, with activity peaking at the relatively high temperature of 50°C (Figure 6B). However, partial activity (10–13% of maximum) was observed at temperatures below 10°C. The enzyme was also found to be active near neutral pH in the 6.0-8.0 pH range, with optimum activity observed at pH 6.5 (Figure 6B). Based on these results, the optimum conditions for β-galactosidase activity were determined to be 4.0 M NaCl or KCl, pH 6.5, and 50°C.

### Effect of organic solvents on the activity and stability of β-galactosidase

**Figure 7:**
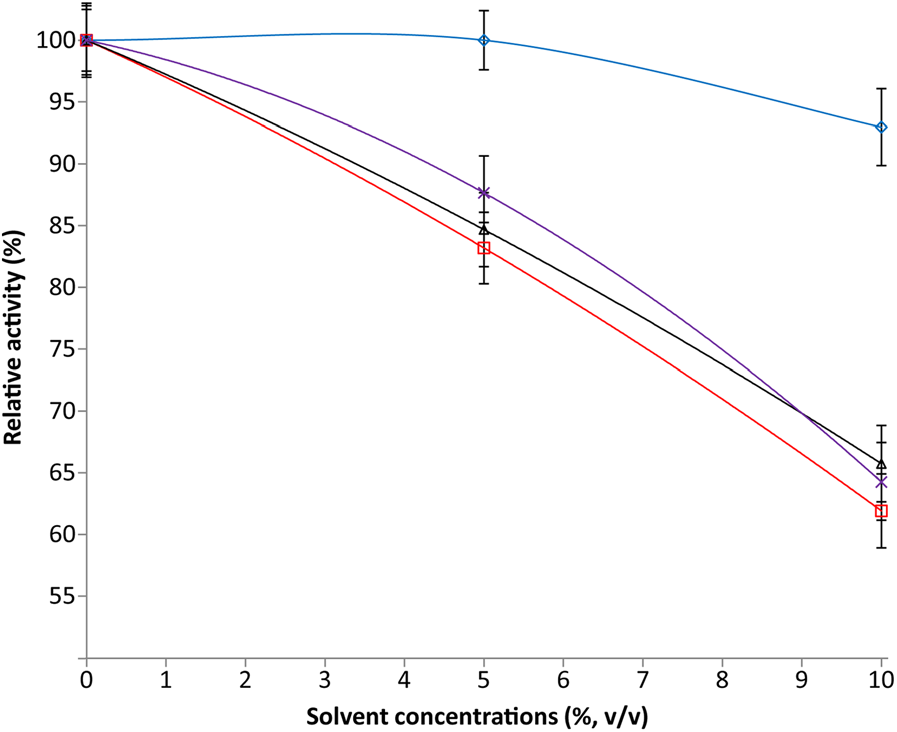
**Effect of organic solvents on purified *H. lacusprofundi *β-galactosidase activity.** The enzyme assay was done in presence of 0, 5, or 10% organic solvents. methanol (blue, ◊), ethanol (red, □), *n-*butanol (black, ∆), isoamyl alcohol (purple, x). Values are the average of triplicate experiments, with standard deviation shown as error bars.

The effect of addition of organic solvents on the activity and stability of the *H. lacusprofundi* β-galactosidase was studied next. Activity was determined in 5 or 10% (v/v) solutions of methanol, ethanol, *n-*butanol and isoamyl alcohol in water with 2.0 M KCl, close to saturation in these aqueous alcohol solutions. There was very little (<5%) reduction of the enzyme activity in the presence of methanol while in the presence of ethanol, *n-*butanol, and isoamyl alcohol, somewhat greater reduction in activity, 30–35%, was recorded (Figure 7). Solvent stability of β-galactosidase was investigated by incubation with methanol, ethanol, *n-*butanol and isoamyl alcohol for 3 h (final concentrations 10 and 20%, v/v) (data not shown). There was relatively little loss of enzyme activity in the presence of the organic alcohols, as little as 3–4% after the first hour and 25–30% after 3 hours. These results show that the *H. lacusprofundi* β-galactosidase enzyme is able to function for significant lengths of time even in the presence of substantial concentrations of organic solvent-water mixtures.

## Discussion

We have established that the glycoside hydrolase GH-42 family *bga* gene in the cold-adapted Antarctic haloarchaeon *H. lacusprofundi* produces a β-galactosidase protein that is polyextremophilic. In order to characterize the salient properties of this novel enzyme, we developed a cold-inducible, cold shock protein *csp*D2 gene promoter-based expression plasmid in the genetic model system, *Halobacterium* sp. NRC-1, and overexpressed the *H. lacusprofundi bga* gene. A high level of active β-galactosidase protein was produced in *Halobacterium* sp. NRC-1 and purified by gel filtration and hydrophobic interaction chromatography, and its identity was established by LC-MS/MS, SDS-PAGE, and ONPG hydrolysis. We found that the β-galactosidase enzyme was overexpressed 20-fold, and displayed very similar properties, with optimal activity at nearly saturated concentration of salts, 4 M NaCl or KCl, and significant measurable activity at low and even subzero temperatures, as well as temperatures above 50°C. Interestingly, we also found that the enzyme was active in the presence of 10–20% organic solvents, including methanol, ethanol, *n*-butanol, and isoamyl alcohol. All together, these findings show a remarkable β-galactosidase displaying enzyme activity at multiple extreme conditions, with significant potential for biotechnological applications [8,42-45]. The enzyme also serves as an excellent model for potential enzymatic activity in extraterrestrial conditions, such as those found on Mars [9].

The *H. lacusprofundi* β-galactosidase is one of few polyextremophilic enzymes to be purified and studied in detail [46]. In the past, a barrier to such studies has been the requirement of high salt concentrations to obtain enzyme activity during overexpression in a foreign host, since low ionic strength conditions generally lead to misfolding or inactivation [8,47]. To avoid problems overproducing active *H. lacusprofundi* β-galactosidase in common non-halophilic hosts such as *E. coli*, we chose the haloarchaeal host, *Halobacterium* sp. NRC-1, for overexpression. This was anticipated to be an optimal host due to its high internal salt concentration, viability at low temperatures, completely sequenced genome, lack of endogenous β-galactosidase, and many available microbiological and molecular genetic tools [12,24,25,38]. In order to maximize expression of the cold-active β-galactosidase in *Halobacterium* sp. NRC-1, we introduced a cold-active promoter for the cold shock protein gene, *csp*D2, into a haloarchaeal expression vector [40]. The *csp*D2 gene was selected based on previous transcriptomic studies of *Halobacterium* sp. NRC-1 [33]. The combination of high salinity and low temperature induction in NRC-1 led to the successful programmed production of high amounts of active β-galactosidase, nearly 20-fold more than in its natural host.

Another challenge in studies of haloarchaeal proteins has been the development of a purification method, as a result of interference of many analytical and chromatographic techniques by high salinity levels [47]. For purification of the *H. lacusprofundi* enzyme overexpressed in *Halobacterium* sp. NRC-1, we used a combination of ion exclusion chromatography and hydrophobic interaction chromatography, since both methods are distinguished by their ability to be applied at high salinity. It has been observed that proteins with hydrophobic “patches” on their surface tend to bind hydrophobic matrixes, a process that is facilitated by high salt concentrations [48,49]. Similarly, ion exclusion chromatography has been successful over a wide range of ionic strength buffers, even those at high salinity [50]. In the past, ion-exchange chromatography was also used for purification of a mesophilic halophilic β-galactosidase from the haloarchaeon, *Haloferax alicantei*; however, the temperature profile for this enzyme was not reported [14].

For the *H. lacusprofundi* β-galactosidase, purity was confirmed by the presence of a highly prominent band on SDS-PAGE, and its identity was verified by LC-MS/MS analysis and enzymatic breakdown of the chromogenic substrates. As previously observed for highly acidic haloarchaeal proteins, anomalous migration was expected during electrophoreses in SDS-PAGE gels, because of the binding of detergents with electrostatic and hydrophobic interactions slows the rate of migration [41,51]. Consequently, the *bga* polypeptide displayed an anomalous molecular mass of ca. 100 kDa, about 28% higher than the predicted molecular mass of 78.06 kDa. However, the protein identity was validated by LC-MS/MS, with 13 peptides covering 14% of the predicted amino acid sequence. The breakdown of the chromogenic substrates, X-gal on agar plates by *Halobacterium* sp. NRC-1(pMC2) colonies, and ONPG by purified enzyme in solution, confirmed that the β-galactosidase was enzymatically active.

The purified *H. lacusprofundi* β-galactosidase was found to be extremely halophilic and retained partial activity at cold temperature and surprisingly also at elevated temperature. It exhibited maximal activity in the presence of 4.0 M NaCl/KCl, which are similar to the intracellular ionic composition observed in other haloarchaea [24,52]. Halophilic enzymes usually feature an increase in the number of charged amino acids, especially acidic residues at the protein surface and the negative surface charge is critical to their solubility and prevents aggregation at high salt concentrations [8,37,47]. Although the temperature optimum was 50°C for both crude extracts and purified β-galactosidase from *Halobacterium* sp. NRC-1 (pMC2), the relative enzyme activity at 60°C was slightly higher for the crude extract. A reason for the observed difference could be that the purified enzyme was used without prior addition of stabilizer. The purified β-galactosidase showed a substantial fraction of activity, nearly 13% at 10°C and 10%, at 4°C. Similar temperature characteristics have been previously reported for other cold-active family 42 β-galactosidases from *Arthrobacter* sp. 32c [26] and *Carnobacterium* sp. BA [53], indicating that extremophilic enzymes frequently function suboptimally under physiological conditions. The pH optimum of β-galactosidase was near neutral, similar to other family 42 β-galactosidases [16,18,26] and in contrast to family 2 β-galactosidases, which are optimally active in alkaline conditions [13,54-56].

In general, non-halophilic enzymes lose most of their activity in the presence of organic solvents [22]. Karan et al. [57] have recently reported that commercial enzymes lose a significant fraction of activity under similar conditions. The *H. lacusprofundi* β-galactosidase, in contrast, was found to be remarkably active and stable in aqueous organic solvent mixtures. In previous work, another cold-adapted β-galactosidase from Antarctic bacterium *Arthrobacter* sp. 32c was also found to be active in similar ethanol concentrations (≤ 20%) [26]. A protease from halophilic archaeon *Natrialba magadii* was found to be active and stable in aqueous-organic solvent mixtures containing 1.5 M NaCl and dioxane [58]. In other studies, halophilic enzymes have been reported to be active and stable in biphasic solutions of water and hydrocarbon organic solvents, such as benzene. These include an amylase of a haloarchaeon, *Haloarcula* sp. strain S-1 [59], and a protease from the halophilic bacterium, *Geomicrobium* sp. EMB2 [49]. These studies indicate that organic solvent stability is a general property of halophilic enzymes, owing to their ability to work at low water activity. However, this is the first report of retention of high levels of enzyme activity in short and long chain alcohols, which reflect the polyextremophilic character of the enzyme.

Polyextremophilic characteristics make the *H. lacusprofundi* β-galactosidase an ideal candidate for industrial and biotechnological uses. For example, the solvent stability of *H. lacusprofundi* β-galactosidase can be utilized for synthesis of oligosaccharides in a similar manner to past studies, but with the added benefit of cold activity and halophilicity. Maugard et al. [60] have exploited a solvent stable β-galactosidase for the synthesis of galacto-oligosaccharides from lactose. Recently, Bridiau et al. [61] reported a *tert*-butanol stable β-galactosidase from *Bacillus circulans* that synthesized *N*-acetyl-lactosamine in hydro-organic media. Vic et al. [62] have also reported the synthesis of 2-hydroxybenzyl β-D-glucopyranoside using β-galactosidase in a *tert*-butanol-water mixture.

The *H. lacusprofundi* β-galactosidase gene is located in a genomic region encoding proteins for binding, uptake, and catabolism of sugars. Since the environment of Deep Lake does not contain lactose, the β-galactosidase gene and surrounding gene cluster are likely to be involved in degradation and utilization of other carbohydrates, such as plant oligo- and polysaccharides [21]. These genes reflect a substantial resource for directing the conversion of biomass into valuable commodities, such as biofuels. The properties described for the purified β-galactosidase are likely to be useful for the development and use of haloarchaea in biotechnology. Moreover, our ability to genetically manipulate and shuttle these and other genes represents a substantial resource for the future.

Halophilic Archaea offer an incomparable resource of polyextremozymes which are active and stable in high concentrations of salt, a broad range of temperatures, and organic solvents. At high salinity, water is sequestered in hydrated ionic structures, limiting the availability of free water molecules for protein hydration [37]. Since halophilic enzymes are adapted to function at high salt concentrations, they are also found to be active at low water availability. Such conditions are also encountered by enzymes in cold temperatures due to the freezing of water molecules and consequent formation of structured ice-like lattices [8]. Therefore, structure-function studies of cold-active haloarchaeal enzyme, including *H. lacusprofundi* β-galactosidase, are likely to provide further insights into enzyme catalysis under water limiting conditions, which are likely to enhance their applications in biotechnology.

Since *H. lacusprofundi* survives in the Antarctic Deep Lake, one of the coldest and most extremely saline environments from which microbes have been cultured, the unusual and unique properties of its enzymes are of general interest to astrobiologists [5,9]. Such environments may be analogs of regions of Earth’s sister planet, Mars, where the potential for biological activity is of intense interest. Images from the Mars Reconnaissance Orbiter showed evidence for seasonal emergence of liquid flows in summer, findings consistent with briny liquid water emerging from underground reservoirs on the planet [63]. The Jovian satellite Europa is covered by frozen water-ice and the presence of liquid water beneath the surface has been hypothesized [64,65]. Further studies of model enzymes like β-galactosidase and the polyextremophilic microbe *H. lacusprofundi* are likely to provide greater insights into how life may be able to cope with challenging conditions on other worlds.

## Conclusions

The genome sequence of *H. lacusprofundi* and directed expression of the β-galactosidase gene in the model haloarchaeon, *Halobacterium* sp. NRC-1, provided a polyextremophilic enzyme for investigation. The purified enzyme was found to be active in high salt concentrations (4–4.5 M NaCl and KCl) and both low (<10°C) and high (>50°C) temperatures, as well as in 10–20% aqueous-organic alcohol solutions. The enzyme provides a platform for improving our understanding of biocatalysis under extreme water limiting conditions and for potential applications in synthetic chemistry. *H. lacuprofundi* encoded proteins and the haloarchaeal genetic system represent significant resources for biotechnology. The system provides the basis for better understanding of life in a perennially cold, hypersaline environment, with relevance to life elsewhere.

## Competing interests

The authors declare that they have no competing interests.

## Authors’ contributions

RK purified and biochemically characterized the enzyme, MDC constructed the expression vector, PD conducted the genetic and genomic analysis, and SD assisted with genomic analysis and finalized the manuscript preparation, with assistance from RK, PD, and MDC. All authors read and approved the final manuscript.
